# The Effect of War on STEMI Incidence: Insights from Intensive Cardiovascular Care Unit Admissions †

**DOI:** 10.3390/jcm13051356

**Published:** 2024-02-27

**Authors:** Ranel Loutati, Sharon Bruoha, Louay Taha, Mohammad Karmi, Nimrod Perel, Tomer Maller, Itshak Amsalem, Rafael Hitter, Nir Levi, Netanel Zacks, Maayan Shrem, Motaz Amro, Mony Shuvy, Michael Glikson, Elad Asher

**Affiliations:** 1 Jesselson Integrated Heart Center, The Eisenberg R&D Authority, Shaare Zedek Medical Center, The Faculty of Medicine, Hebrew University of Jerusalem, Jerusalem 9112002, Israel; ranellout@gmail.com (R.L.); louayt@szmc.org.il (L.T.); mkarmi@szmc.org.il (M.K.); nperel@szmc.org.il (N.P.); tomerma@szmc.org.il (T.M.); itshakam@szmc.org.il (I.A.); rafaelhi@szmc.org.il (R.H.); levin@szmc.org.il (N.L.); zacks.netanel@gmail.com (N.Z.); motaza@szmc.org.il (M.A.); monysh@szmc.org.il (M.S.); mglikson@szmc.org.il (M.G.); 2 Department of Cardiology, Barzilai Medical Center, and The Ben-Gurion University of the Negev, Ashkelon 7830604, Israel; sharonitob@gmail.com

**Keywords:** ICCU, ACS, STEMI

## Abstract

(1) **Background**: The impact of armed conflicts on public health is undeniable, with psychological stress emerging as a significant risk factor for cardiovascular disease (CVD). Nevertheless, contemporary data regarding the influence of war on CVD, and especially on acute coronary syndrome (ACS), are scarce. Hence, the aim of the current study was to assess the repercussions of war on the admission and prognosis of patients admitted to a tertiary care center intensive cardiovascular care unit (ICCU). (2) **Methods**: All patients admitted to the ICCU during the first three months of the Israel–Hamas war (2023) were included and compared with all patients admitted during the same period in 2022. The primary outcome was in-hospital mortality. (3) **Results**: A total of 556 patients (184 females [33.1%]) with a median age of 70 (IQR 59–80) were included. Of them, 295 (53%) were admitted to the ICCU during the first three months of the war. Fewer Arab patients and more patients with ST-segment elevation myocardial infraction (STEMI) were admitted during the war period (21.8% vs. 13.2%, *p* < 0.001, and 31.9% vs. 24.1%, *p* = 0.04, respectively), whereas non-STEMI (NSTEMI) patients were admitted more frequently in the pre-war year (19.3% vs. 25.7%, *p* = 0.09). In-hospital mortality was similar in both groups (4.4% vs. 3.4%, *p* = 0.71; HR 1.42; 95% CI 0.6–3.32, *p* = 0.4). (4) **Conclusions**: During the first three months of the war, fewer Arab patients and more STEMI patients were admitted to the ICCU. Nevertheless, in-hospital mortality was similar in both groups.

## 1. Introduction

Armed conflicts pose hazards to both short-term and long-term health outcomes [1,2]. In the immediate aftermath, the chaos of conflict presents logistical challenges, hindering efficient patient referral systems and exacerbating the difficulties in accessing timely medical assistance. Furthermore, heightened emotional stress and anxiety experienced by individuals in conflict zones can lead to increased disease rates and further impede healthcare access [3]. Looking ahead, the enduring repercussions of armed conflicts extend far beyond the battlefield. Increased military expenditures divert resources away from healthcare systems, exacerbating existing challenges and straining the capacity to address the growing healthcare burden stemming from both physical and psychological injuries. Moreover, the pervasive nature of conflict-induced stress may give rise to maladaptive behaviors, including alcohol, tobacco, and illicit drug use, further compounding the health challenges faced by affected populations and perpetuating the decline in overall health outcomes [1,2,3,4,5]. The impact of natural disasters and pandemics on cardiovascular disease (CVD) has been extensively studied [6,7,8]. However, research on the extent and impact of wars on CVD is scarce [9]. Notably, areas affected by earthquakes or populations engaged in armed conflicts have shown an increase in the incidence of acute coronary syndrome (ACS), along with higher rates of in-hospital complications and, subsequently, a more unfavorable prognosis. These adverse outcomes are attributed to delayed presentation and reduced access to urgent reperfusion treatment [9,10,11,12,13,14]. In modern armed conflicts, several characteristic features, such as low intensity, prolonged duration, and heightened levels of stress and psychological distress, are observed. These factors are further exacerbated by exposure through social media and psychological terror, contributing to a complex interplay between war-related stressors and CV health [2,3]. However, there is a paucity of contemporary data regarding the impact of these modern conflicts on cardiovascular health, especially on acute coronary syndrome (ACS). Therefore, the aim of the current study was to assess the repercussions of modern war on CVD by examining the admission rates and in-hospital course of patients admitted to a tertiary care center intensive cardiovascular care unit (ICCU) during the first three months of the Israel–Hamas 2023 war and comparing them to the corresponding period in the previous year. 

## 2. Methods

### 2.1. Study Population

This study is a single-center observational cohort study that was performed in the ICCU of Shaare Zedek Medical Center, a tertiary referral hospital and one of the two largest medical centers in Jerusalem which treats more than 1000 patients with various CV diseases every year. The study population consisted of non-selected consecutive patients admitted to the ICCU during the first three months of the Israel–Hamas conflict (7 October 2023–7 January 2024) who were prospectively enrolled. The first three months of the war were studied as this period witnessed the most intense acts of war. The second group of patients included in this study are those who were admitted to the ICCU during the corresponding period in 2022 (7 October 2022–7 January 2023), who were retrospectively included for comparison. All patients admitted during the study period were included. 

### 2.2. Data Collection

Data were anonymously documented in the ICCU by the local coordinator and prospectively submitted into an electronic case report form (eCRF). Data were checked for accuracy and out-of-range values by the coordinating unit, and any case of inconsistency was addressed. Demographic data, presenting symptoms, comorbid conditions, and physical examination were systematically recorded. Laboratory, imaging, angiographic results, and clinical course data were collected as well [15]. Patients were divided into two groups based on the year of admission, with patients admitted between 7 October 2022 and 7 January 2023 serving as the reference group.

The institutional review board approved the study based on strict maintenance of participants anonymity by de-identifying the participants during database analysis. No individual consent was obtained. Moreover, the authors have no conflicts of interest to declare. No funding was applied to the study.

### 2.3. Study Outcomes

The primary outcome was in-hospital mortality, which was recorded as an outcome for every patient. Additional outcomes that were studied are differences in demographics, baseline characteristics, main diagnosis, treatments, and in-hospital complications. Treatments that were compared included coronary angiography and percutaneous coronary intervention (PCI), pacemaker or implantable cardioverter defibrillator (ICD) implantations, and advanced therapies such as mechanical ventilation and extra-corporeal membrane oxygenation (ECMO). In-hospital complications encompass shock (of any type), acute renal failure (ARF), significant bleeding (as indicated by Bleeding Academic Research Consortium (BARC) types 3 and 5), and vascular complications.

### 2.4. Statistical Analysis

Continuous variables were expressed as mean ± standard deviation if normally distributed or median with interquartile range if skewed. Categorical variables were presented as frequency (%). Differences in demographics, baseline characteristics, main diagnosis, treatment, and complications between the two groups of patients were studied. A comparison of means was performed using Student’s *t*-test or Mann–Whitney U-test where appropriate. Statistical comparison of the differences in categorical data between the two groups was performed using the chi-square test or the Fisher’s exact test. Cox proportional hazards regression was used to compare in-hospital mortality in patients who were admitted during the war with patients who were admitted during the corresponding period in the preceding year. Hazard ratios (HRs) and corresponding 95% confidence intervals (CIs) for the association between in-hospital mortality and the war period are presented. All analyses were performed using R software version 3.4.4 (R Foundation for Statistical Computing). An association was considered statistically significant for a two-sided *p*-value of less than 0.05.

## 3. Results

### 3.1. Baseline Characteristics

The study population comprised a total of 556 patients. The median age was 70 years old [interquartile range (IQR): 59–80], and 184 (33.1%) were female. A total of 295 (53%) patients were admitted during the war, while 261 (47%) patients were admitted in the preceding year. There were no statistically significant differences between the periods of admissions regarding age, sex, and comorbidities. However, fewer Arab patients were admitted during the war (*n* = 57 [21.8%] vs. 39 [13.2%], *p* < 0.001). Baseline characteristics of ICCU patients stratified by admission period are presented in Table 1.

### 3.2. Main Diagnosis on Admission

**Figure 1:**
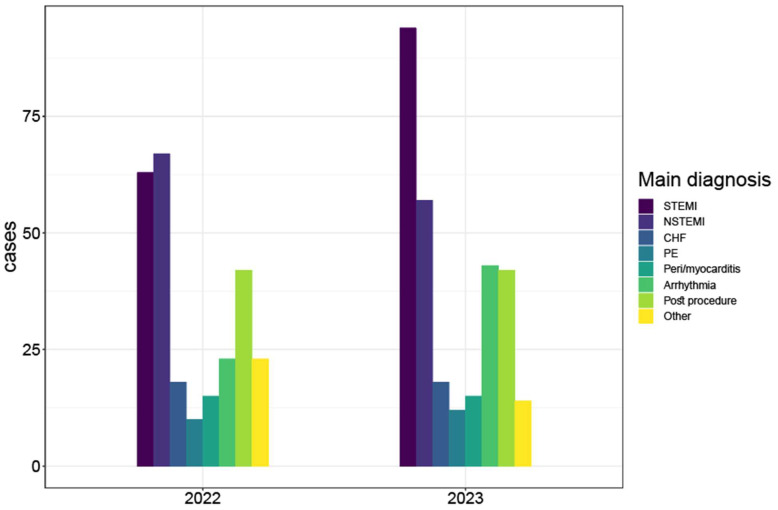
Bar plot of cases by year grouped by main diagnosis. This bar plot demonstrates the relative portion of the main diagnoses in the ICCU in each of the periods, showing that STEMI was more prevalent during the war period (2023). CHF = congestive heart failure; NSTEMI = non ST-elevation myocardial infraction; PE = pulmonary embolism; STEMI = ST segment elevation myocardial infraction.

Main diagnoses on admission are presented in Figure 1 and Table 2. Patients who were admitted during the war had higher rates of STEMI and lower rates of NSTEMI as compared with the pre-war year (31.9% vs. 24.1%, *p* = 0.04, and 19.3% vs. 25.7%, *p* = 0.09, respectively). Additionally, arrhythmias, including both tachyarrhythmias and bradyarrhythmias, were more common as the main diagnosis on admission during the wartime (14.6% vs. 8.8%, *p* = 0.048).

### 3.3. Interventions and Complications during ICCU Admission

Interventions and complications are summarized in Table 3. Coronary angiography with and without intervention was performed at similar rates during both the war period and the pre-war year (153 [51.9%] vs. 148 [56.7%], *p* = 0.29). Similarly, the rates of mechanical ventilation, intra-aortic balloon pump (IABP), Impella, and extra-corporeal membrane oxygenation (ECMO) utilization, were also similar in both periods. There was no between-group difference in the overall complication rate during admission (13.9% vs. 14.9% during the war vs. the pre-war year, *p* = 0.85). 

### 3.4. In-Hospital Mortality

**Figure 2:**
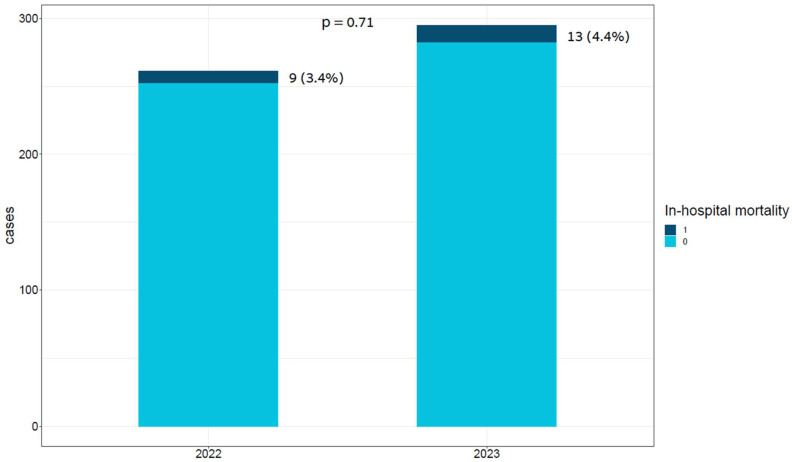
Bar plot of cases by year and in-hospital mortality status. This bar plot demonstrates the relative portion of in-hospital mortality in each period, presenting no significant difference between the periods.

In-hospital mortality was similar in both groups [13 (4.4%) vs. 9 (3.4%), *p* = 0.71], as presented in Figure 2. A univariate Cox regression model demonstrated that patients admitted during the war did not have an increased risk of in-hospital mortality compared to those who were admitted in the pre-war year (HR 1.42; 95% CI 0.6–3.32, *p* = 0.4).

## 4. Discussion

The main findings of our study were as follows: (1) during the war period, the absolute number of patients admitted to the ICCU was higher than in the pre-war period; (2) fewer Arab patients were admitted during the period of conflict; (3) higher rates of STEMI and lower rates of NSTEMI admissions occurred during the war. Lastly, although the in-hospital mortality rate was numerically higher during the period of conflict, it did not reach statistical significance.

Armed conflicts are associated with increased CV morbidity and mortality [9]. However, the higher incidence of heart diseases does not necessarily translate into higher admission rates. Shortages in healthcare resources and increased perception of danger due to ongoing acts of violence can result in a lower tendency of civilians to seek medical care [16,17]. This was highly evident during the COVID-19 pandemic, as hospital admissions for all major non-COVID-19 disease groups decreased [18]. Nevertheless, in our analysis, the number of patients admitted to the ICCU was higher compared to the pre-war period.

Surprisingly, we observed a concerning and notable decrease in ICCU admissions among Arabs compared to Jews, indicating a reduced inclination to seek medical care. It is important to note that Arabs in Israel exhibit a higher prevalence of CV risk factors, including diabetes mellitus (DM), increased body mass index (BMI), altered lipid profiles, and higher smoking rates. They also experience a greater incidence of ischemic heart disease at a younger age and have higher rates of all-cause mortality among patients with ischemic heart disease compared with Jews [19]. Therefore, the increased incidence of cardiac events related to mental stress, as well as lower propensity for seeking medical care, could lead to poorer health outcomes specifically in this ethnic group, particularly during periods of conflict. This phenomenon might be explained by Arabs’ reluctance to pursue medical care due to fears of potential exposure to acts of war, such as rocket barrages and/or concerns over potential confrontations with Jews stemming from the adverse national sentiment due to the Israel–Hamas war. Importantly, the observed lower admission rates of Arabs, coupled with an increased number in overall ICCU admissions, suggest that our analysis may underestimate the actual magnitude of the change in cardiac events incidences during the conflict. 

The detrimental impact of emotional stress on cardiovascular health is multifaceted and complex [20], involving a cascade of physiological responses that can ultimately lead to adverse cardiovascular events. During periods of armed conflict, individuals are exposed to intense and prolonged emotional stressors, which can significantly disrupt their physiological equilibrium and increase their susceptibility to cardiovascular diseases. These stressors trigger a series of neurohormonal changes, including sympathetic overstimulation and the dysregulation of stress hormone pathways, which, in turn, contribute to alterations in immune–inflammatory responses, endothelial dysfunction, and behavioral patterns [21,22]. Consequently, armed conflicts are linked to increased incidence of DM, raised blood pressure and cholesterol level, and increased alcohol and tobacco use [9]. Moreover, studies have consistently shown a marked increase in the incidence of acute myocardial infarction and complex coronary disease among populations exposed to conflict situations [12,23]. This heightened risk of cardiovascular events during times of conflict is further exacerbated by individual perceptions of stress, with those reporting higher levels of perceived stress experiencing a more pronounced increase in acute coronary syndrome incidence. Thus, the relationship between armed conflicts and cardiovascular health is intricately linked to the interplay between psychological stressors and physiological responses, underscoring the importance of addressing both mental and physical health needs in conflict-affected populations [24,25].

While the mechanisms by which stress precipitates ACS are not entirely elucidated, experiments show that stress-induced leukocyte recruitment in atherosclerotic plaques and increased fibrous cap extracellular matrix breakdown are central to promoting plaque destabilization, and particularly plaque rupture, the principal underlying mechanism in STEMI [26,27]. However, studies reveal that acute stress is associated with a significant increase in all ACS spectra [28]. In addition, stress-induced catecholamines release may cause myocardial injury in the absence coronary thrombosis by triggering, for instance, microvascular dysfunction or coronary vasospasm [29]. Finally, we also found a strong relationship between acute stress reactions and arrhythmias (14.6% vs. 8.8%, *p* = 0.048). Cardiac autonomic dysregulation appears to alter myocardial electrophysiological properties, thereby promoting cardiac arrhythmias following psychological stress [28,30,31].

We believe that the lower rate of NSTEMI admissions is linked to the ongoing war and the high perception of danger. Consequently, older individuals with less severe infarcts might endure the discomfort and choose to avoid visiting the hospital. Indeed, the threshold for seeking medical attention rises in times of crisis. Furthermore, individuals with poor health are more likely to delay care than those with good health. Not surprisingly, delayed or avoided medical care are associated with poor outcomes [32,33].

### Study Limitations

Our study possesses several limitations: (1) It was conducted within a singular tertiary-care ICCU, inherently prone to referral bias. Nonetheless, this ICCU center annually provides care for over 1000 patients. (2) This analysis focused solely on short-term mortality rates and was confined to the initial three months of the conflict. (3) Our medical facility is situated at a relative distance from the active battle zone and encountered fewer rocket attacks during the war in contrast to regions near the combat area. (4) Our analysis primarily centered on overall in-hospital mortality rather than cardiovascular-specific mortality, although mortality patterns in Israel closely mirror those of the European Union, where cardiovascular deaths rank second following cancer [34]. (5) Potential confounding variables, including the elapsed time between symptom onset and acute coronary syndrome (ACS) diagnosis, Killip class upon presentation, New York Heart Association (NYHA) functional class, and B-type natriuretic peptide (BNP) levels, were not measured and were therefore not available for analysis. (6) While independent associations have been identified, the study’s design precludes establishing causality. 

## 5. Conclusions

Consistent with prior research, our findings reveal a robust correlation between cardiovascular disease (CVD) and periods of armed conflict, potentially attributable to the psychological stress inherent in such circumstances. Notably, we observed a concerning trend of reduced medical care-seeking behavior among Arabs, which may contribute to unfavorable outcomes. Furthermore, heightened rates of ST-elevation, myocardial infarction (STEMI), and arrhythmias were evident during the conflict period compared to the control phase. Despite these observations, there was no discernible alteration in in-hospital mortality rates during the war.

## Figures and Tables

**Table 1:** Baseline characteristics.

Variable	7 October 2022–7 January 2023 (*N* = 261)	7 October 2023–7 January 2024 (*N* = 295)	*p*-Value
Age (years)	69 (58–79)	70 (59–80)	0.7
Female sex	78 (29.9%)	106 (35.9%)	0.155
BMI (kg/m^2^)	27.3 (24.2–31)	27.4 (24.2–30.4)	0.543
Ethnicity—no. (%)			<0.001
Jews	197 (75.5%)	256 (86.8%)	
Arabs	57 (21.8%)	39 (13.2%)	
Other	7 (2.7%)	0 (0%)	
HTN—no. (%)	156 (59.8%)	155 (52.5%)	0.113
DLP—no. (%)	139 (53.3%)	145 (49.2%)	0.378
DM—no. (%)	98 (37.5%)	102 (34.6%)	0.522
PAD—no. (%)	19 (7.3%)	17(5.8%)	0.58
CHF—no. (%)	39 (14.9%)	49 (16.6%)	0.674
PHTN—no. (%)	5 (1.9%)	25 (8.5%)	<0.001
AFIB—no. (%)	39 (14.9%)	54 (18.3%)	0.344
Prior MI—no. (%)	98 (37.5%)	95 (32.2%)	0.218
Prior CABG—no. (%)	24 (9.2%)	32 (10.8%)	0.614
COPD—no. (%)	27 (10.3%)	18 (6.1%)	0.09
CKD—no. (%)	31 (11.9%)	30 (10.2%)	0.612
Prior stroke—no. (%)	17 (6.5%)	15 (5.1%)	0.59
Smoking—no. (%)	74 (28.4%)	66 (22.4%)	0.171
Family History of CAD—no. (%)	18 (6.9%)	23 (7.8%)	0.808

Values are the median (interquartile range: [Q1–Q3]) for continuous variables and the number of occurrences (frequency %) for categorical variables. AFIB = atrial fibrillation; BMI = body mass index; CABG = coronary artery bypass graft surgery; CAD = coronary artery disease; CHF = congestive heart failure; CKD = chronic kidney disease; COPD = chronic obstructive pulmonary disease; DLP = dyslipidemia; DM = diabetes mellitus; HTN = hypertension; MI = myocardial infraction; PAD = peripheral artery disease; PHTN = pulmonary hypertension.

**Table 2:** Main diagnosis by period.

	7 October 2022–7 January 2023 (*N* = 261)	7 October 2023–7 January 2024 (*N* = 295)	*p*-Value
STEMI	63 (24.1%)	94 (31.9%)	0.04
NSTEMI	67 (25.7%)	57 (19.3%)	0.09
CHF	18 (6.9%)	18 (6.1%)	0.836
PE	10 (3.8%)	12 (4.1%)	1
Peri/myocarditis	15 (5.7%)	15 (5.1%)	0.87
Arrhythmia	23 (8.8%)	43 (14.6%)	0.048
Post procedure	42 (16.1%)	42 (14.2%)	0.64
Other	23 (8.8%)	14 (4.7%)	0.08

Values are number of occurrences (frequency %). CHF = congestive heart failure; NSTEMI = non-ST-elevation myocardial infraction; PE = pulmonary embolism; STEMI = ST segment elevation myocardial infraction.

**Table 3:** Interventions and complications during admission.

	7 October 2022–7 January 2023 (*N* = 261)	7 October 2023–7 January 2024 (*N* = 295)	*p*-Value
Interventions			
Coronary angiography—no. (%)	148 (56.7%)	153 (51.9%)	0.29
Urgent PCI (<2 h)—no. (%)	73 (28%)	95 (32.2%)	0.32
PCI—no. (%)	42 (16.1%)	36 (12.2%)	0.23
Pacemaker or ICD implantation—no. (%)	15 (5.7%)	23 (7.8%)	0.43
Blood Transfusion—no. (%)	22 (8.4%)	16 (5.4%)	0.22
CPR—no. (%)	6 (2.3%)	14 (4.7%)	0.187
Mechanical ventilation—no. (%)	32 (12.3%)	35 (11.9%)	0.99
Arterial line—no. (%)	67 (25.7%)	55 (18.6%)	0.05
IABP—no. (%)	5 (1.9%)	5 (1.7%)	0.63
ECMO—no. (%)	2 (0.8%)	2 (0.7%)	1
Complications			
Shock *—no. (%)	11 (4.2%)	19 (6.4%)	0.33
ARF—no. (%)	3 (1.1%)	10 (3.4%)	0.14
Significant Bleeding—no. (%)	14 (5.4%)	17 (5.8%)	0.98
Vascular complication—no. (%)	7 (2.7%)	2 (0.7%)	0.13

Values are the number of occurrences (frequency %) for categorical variables. ARF = acute renal failure; CPR = cardiopulmonary resuscitation; ECMO = extra-corporeal membrane oxygenation; IABP = intra-aortic balloon pump; ICD = implantable cardioverter defibrillator; PCI = percutaneous coronary intervention; * any type of shock.

## Data Availability

The data presented in this study are available on request from the corresponding author (E.A.).
